# Supplementary material for: Smelly communication between haemaphysalis longicornis and infected hosts with indolic odorants: A case from severe fever with thrombocytopenia syndrome virus
Source: PLoS Negl Trop Dis. 2025 Jun 5;19(6):e0013139. doi: 10.1371/journal.pntd.0013139 (PMC12173412; doi:10.1371/journal.pntd.0013139)
Supplement: S1 Table — (DOCX) [file pntd.0013139.s001.docx]

Supplemental Table S1 **Software used in the present study.**

**Table S1: Software used in the present study**

| Analysis | Software | Version | Parameters |
| --- | --- | --- | --- |
| KNN | R (impute) | 1.56.0 | default parameters |
| PCA | R (base package) | 4.1.2 | UV (unit variance scaling) |
| Heatmap | R (ComplexHeatmap) | 2.9.4 | UV (unit variance scaling) |
| Pearson Correlation Coefficients | R (base package) | 4.1.2 | - |
| Correlation diagram between samples | R (corrplot) | 0.92 | - |
| OPLS-DA | R (MetaboAnalystR) | 1.0.1 | log2 transform+ Zero-centered |
| Radar diagram | R (fmsb) | 0.7.1 | - |
| Chord diagram | R (igraph; ggraph) | 1.2.11; 2.0.5 | - |
| Correlate networks | R (igraph) | 1.2.11 | - |
| K-Means | R (base package) | 4.1.2 | UV (unit variance scaling) |
