# Supplementary material for: Smelly communication between haemaphysalis longicornis and infected hosts with indolic odorants: A case from severe fever with thrombocytopenia syndrome virus
Source: PLoS Negl Trop Dis. 2025 Jun 5;19(6):e0013139. doi: 10.1371/journal.pntd.0013139 (PMC12173412; doi:10.1371/journal.pntd.0013139)
Supplement: S2 Table — (DOCX) [file pntd.0013139.s002.docx]

Supplemental Table S2. **Primers and probes for SFTSV screening.**

| SFTSV (S gene) | S-F | GGGTCCCTGAAGGAGTTGTAAA |
| --- | --- | --- |
|  | S-R-3 | TGCCTTCACCAAGACTATCAATGT |
|  | S-Probe | TexasRed-TTCTGTCTTGCTGGCTCCGCGC-BHQ-2 |
