# Supplementary material for: Smelly communication between haemaphysalis longicornis and infected hosts with indolic odorants: A case from severe fever with thrombocytopenia syndrome virus
Source: PLoS Negl Trop Dis. 2025 Jun 5;19(6):e0013139. doi: 10.1371/journal.pntd.0013139 (PMC12173412; doi:10.1371/journal.pntd.0013139)
Supplement: S3 Table — (DOCX) [file pntd.0013139.s003.docx]

S3 Data. **3D homology modeling parameters for NPC2 and OBPL in Swiss-model.**

| Parameters |  | NPC2 | | | | | | | |  | OBPL | | |
| --- | --- | --- | --- | --- | --- | --- | --- | --- | --- | --- | --- | --- | --- |
|  |  | Value | | | Sites | | | | |  | Value | Sites | |
| MolProbity Score |  | 1.99 | | |  | | | | |  | 0.77 |  | |
| Clash Score |  | 11.47 | | | A60HIS, A175PHE, A100PHE, A136 LEU, A58VAL, A60HIS, A62LYS, A66ASP | | | | |  | 0.89 |  | |
| Ramachan ran Favoured |  | 93.65% | | |  | | | | |  | 98.57% |  | |
| Ramachandran Outliers |  | 2.38% | | | A167GLY, A64GLY, A169ASN | | | | |  | 0.00% |  | |
| Rotamer Outliers |  | 0.00% | | |  | | | | |  | 0.00% |  | |
| C-Beta Deviations | | |  | 0 | |  |  | 1 | A147 VAL | | | |  |
| Bad Bonds | | |  | 0/1030 | |  |  | 0/1174 |  | | | |  |
| Bad Angles | | |  | 5/1394 | | A73ASP, (A168ASP-A169ASN), A169ASN, A81HIS, A60HI |  | 1/1590 | (A121GLU-A122PRO), A143PHE, A37AN, A91HIS, A81HIS, A141HIS, A157HS, A72HIS, (A30GLU-A31PRO), A96PHE | | | |  |
