# Supplementary figures and images for: Smelly communication between haemaphysalis longicornis and infected hosts with indolic odorants: A case from severe fever with thrombocytopenia syndrome virus
Source: PLoS Negl Trop Dis. 2025 Jun 5;19(6):e0013139. doi: 10.1371/journal.pntd.0013139 (PMC12173412; doi:10.1371/journal.pntd.0013139)

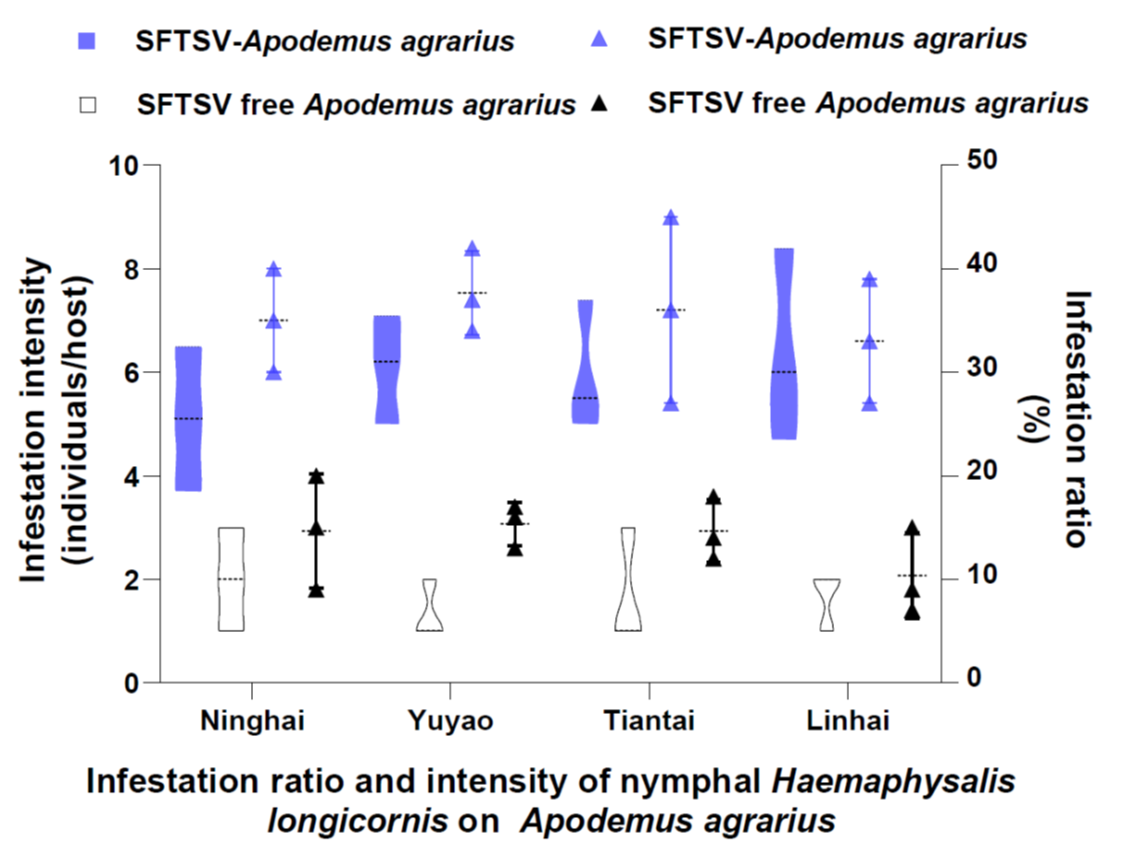

Supplement: S1 Fig — (TIF) [file pntd.0013139.s004.tif]

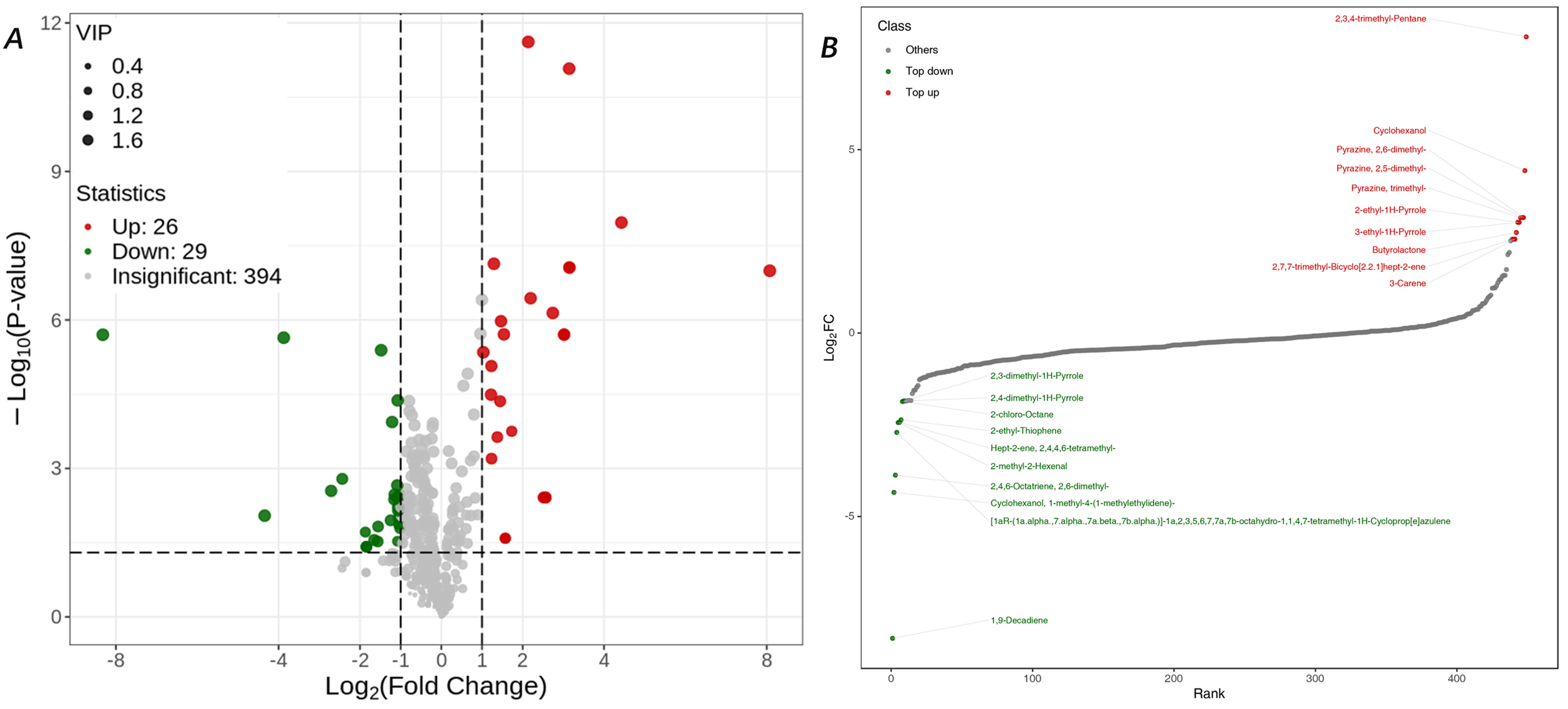

Supplement: S2 Fig — Panel A: volcanic diagram of the identified DVMs; Panel B: identified DVMs and their fold changes. (TIF) [file pntd.0013139.s005.tif]

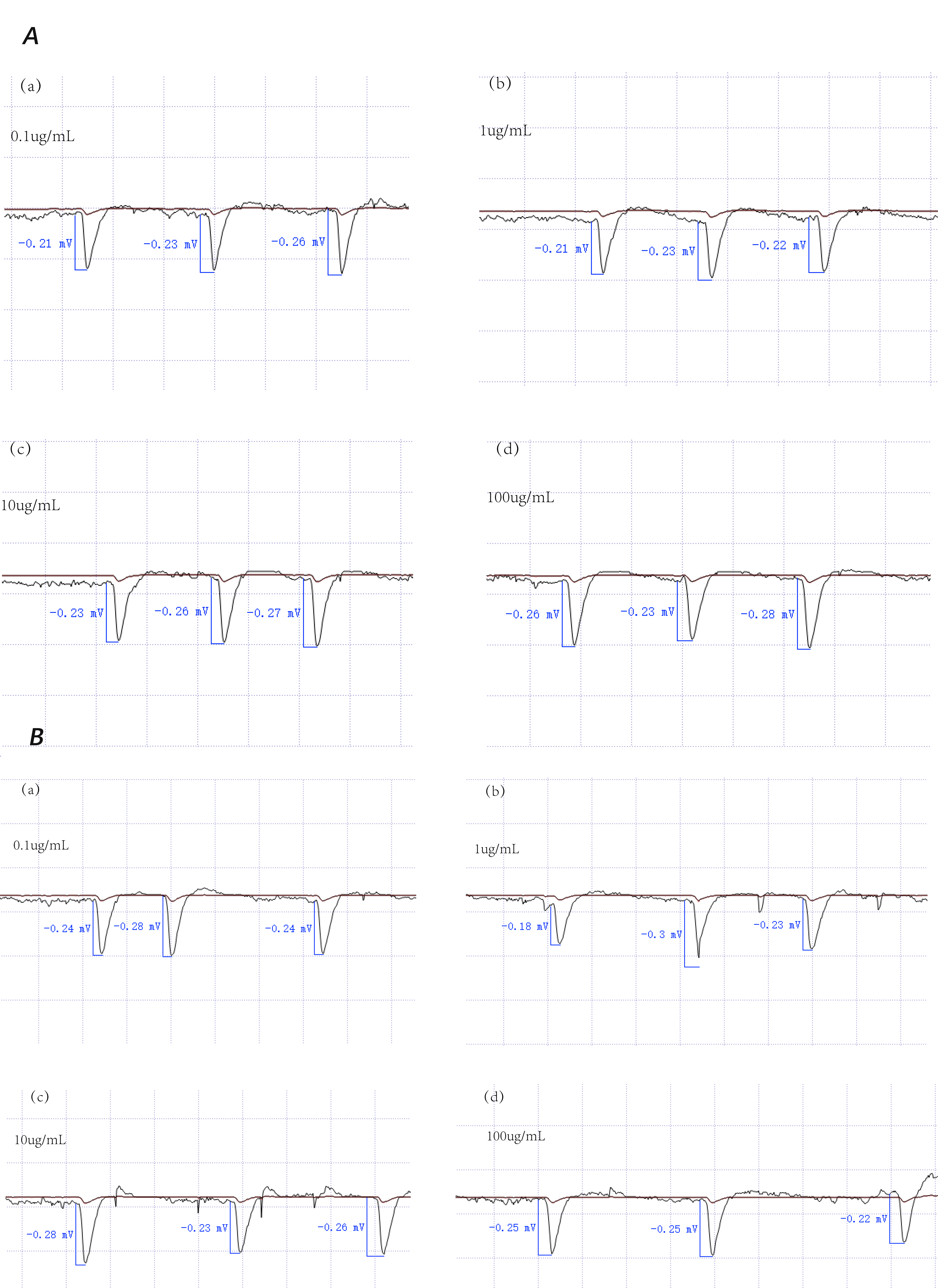

Supplement: S3 Fig — Panel A: indole under different concentrations; Panel B: 3-methylindole under different concentrations. (TIF) [file pntd.0013139.s006.tif]

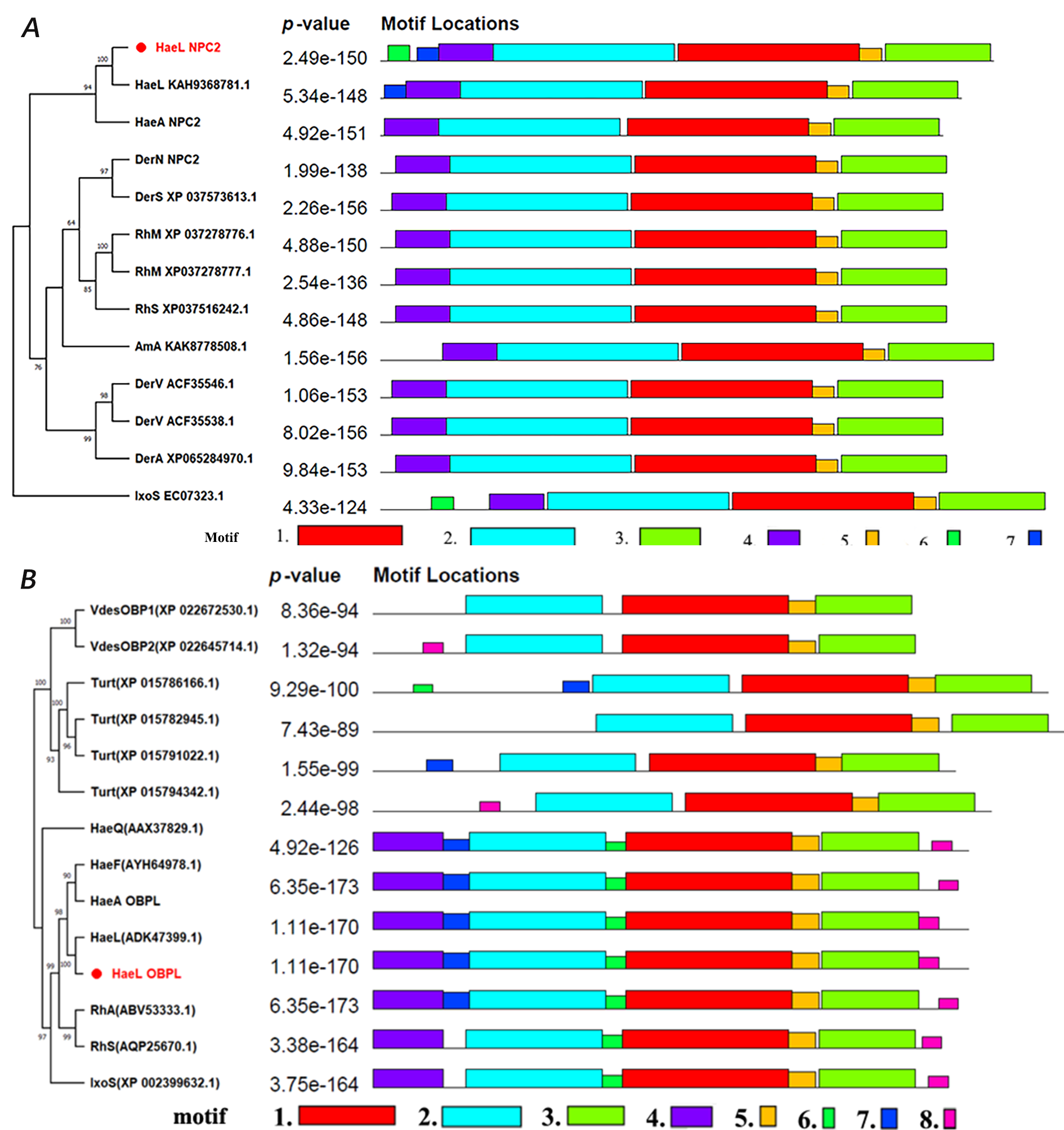

Supplement: S4 Fig — Panel A: NPC2 (HaeL NPC2 accession no. PV029724); Panel B: OBPL (HaeL OBPL accession no. PV029725). (TIF) [file pntd.0013139.s007.tif]

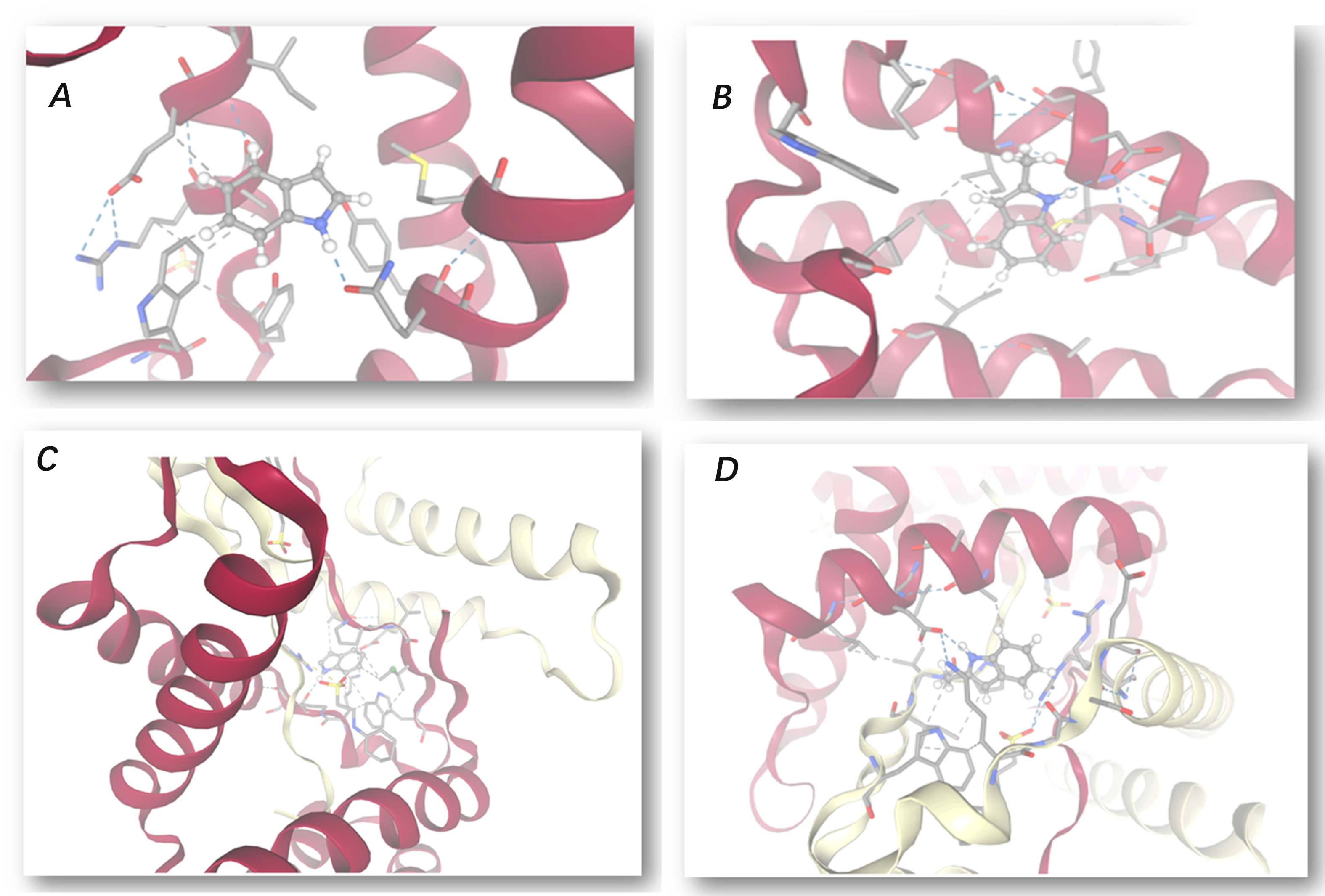

Supplement: S5 Fig — Panel A: NPC2- Indole; Pane B: NPC2–3-methylindole; Pane C: OBPL-Indole; Panel D: OBPL-3-methylindole). (TIF) [file pntd.0013139.s008.tif]

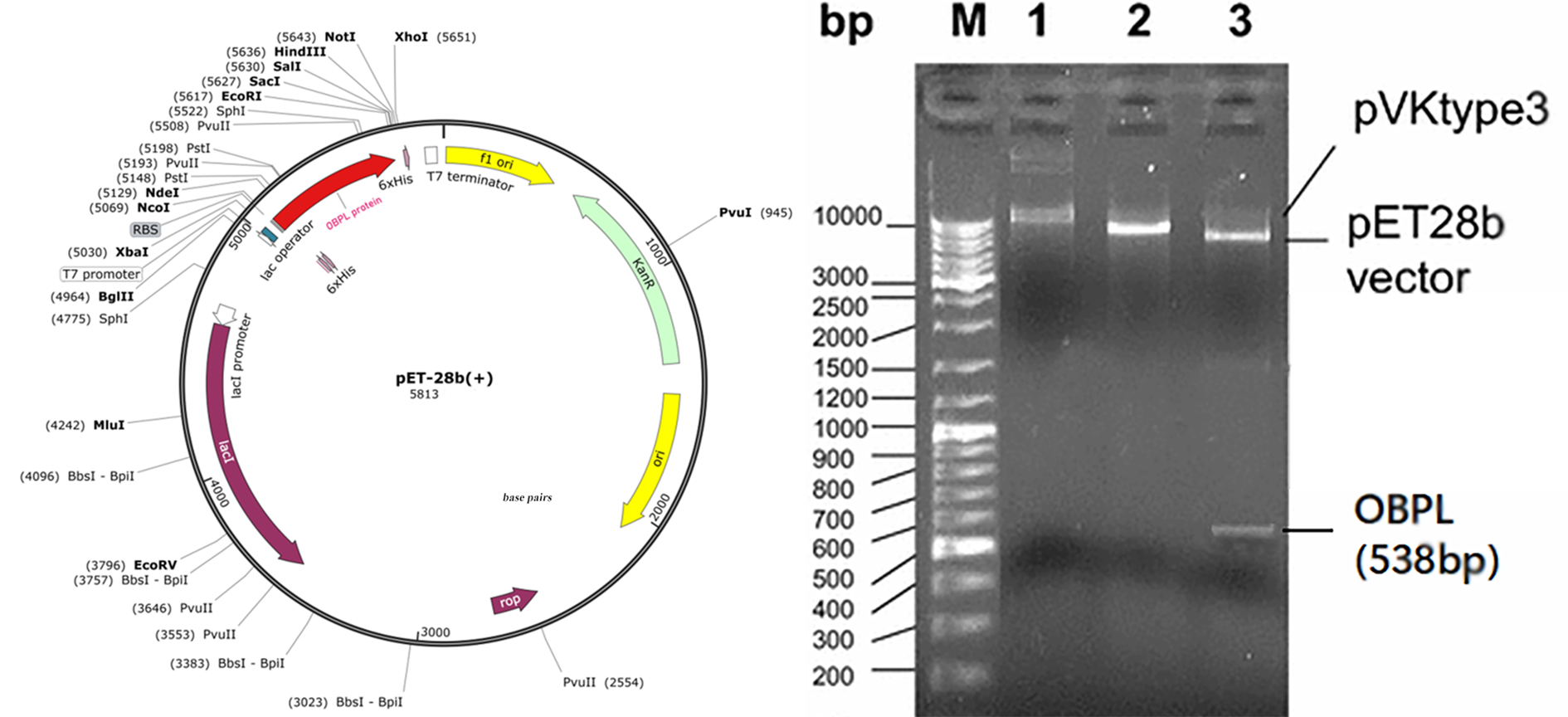

Supplement: S6 Fig — Panel A: the recombined expression plasmid of pET-28b-OBPL; Panel B: the electrophoresis panel of pET-28b-OBPL digested. M: molecular marker, 1–2, the recombined expression plasmid; 3: pVK type 3, blank plasmid pET-28b and target OBPL gene. (TIF) [file pntd.0013139.s009.tif]
