# Supplementary material for: Smelly communication between haemaphysalis longicornis and infected hosts with indolic odorants: A case from severe fever with thrombocytopenia syndrome virus
Source: PLoS Negl Trop Dis. 2025 Jun 5;19(6):e0013139. doi: 10.1371/journal.pntd.0013139 (PMC12173412; doi:10.1371/journal.pntd.0013139)
Supplement: Attachment — Submitted filename: Research evaluation New.docx [file pntd.0013139.s013.docx]

**Research evaluation**

**SMELLY COMMUNICATION BETWEEN *HAEMAPHYSALIS LONGICORNIS* AND INFECTED HOSTS WITH INDOLIC ODORANTS：A CASE FROM SEVERE FEVER WITH THROMBOCYTOPENIA SYNDROME VIRUS**

**Research Summary**

The idea of the research is concerned with an important topic in the field of The biology of living organisms and the use of their behavior in control operations. This article describes a study investigating how the tick *Hae. longicornis* is attracted to hosts infected with Severe Fever with Thrombocytopenia Syndrome Virus (SFTSV). The findings suggest that SFTSV infection in a host leads to characteristic odor changes that attract ticks.

Here's a breakdown of the key points:

**Introduction:**

Ticks need to locate vertebrate hosts for blood feeding, and they use various cues, including odor, to find them.

This study explores how SFTSV infection in a host influences tick attraction through odor changes.

**Methods:**

SFTSV-infected and non-infected mice were used.

Volatile organic compounds (VOCs) emitted by the mice were collected and analyzed.

Tick behavioral responses to these VOCs and specific odor components (indole and 3-methylindole) were tested.

Genes and proteins in ticks potentially involved in odor detection were investigated.

**Results:**

SFTSV infection in mice altered their VOC profiles.

Ticks were more attracted to odors from SFTSV-infected mice compared to non-infected mice.

Indole and 3-methylindole, present in higher levels in infected mice, elicited significant responses in tick olfactory neurons.

Ticks possess odor-binding proteins that may play a role in detecting these specific odor molecules.

**Conclusion:**

SFTSV infection creates a distinct olfactory signature in hosts that attracts Hae. longicornis ticks.

**Research Objective**:

Understand how ticks find hosts infected with specific diseases by studying the odor cues emitted by the infected hosts.

Design repellents or attractants based on these odor cues to control the spread of tick-borne diseases.

**Research Methods**:

Researchers used mice with a weakened immune system (Ifnar-/- mice) infected with SFTSV (Severe Fever with Thrombocytopenia Syndrome Virus) to mimic infected hosts.

Urine and fecal samples were collected from the infected and non-infected mice to identify differences in volatile organic compounds (VOCs) produced by the hosts.

Two specific VOCs, indole and 3-methylindole, were chosen for further investigation due to their potential role in attracting ticks.

Scientists examined the olfactory response of the tick *Hae. longicornis* to these two indole odors using electroantennographic detection (EAD) and Y-tube olfactometer.

To understand the tick's sense of smell, researchers looked at two olfactory proteins (NPC2 and OBPL) found in the tick's forelegs.

They tested how well these proteins bind to indole and 3-methylindole molecules and studied the gene expression of these proteins when exposed to the indole odors.

Overall, the research suggests that *Hae. longicornis* ticks are attracted to the scent of indole produced by SFTSV-infected mice. This finding could be useful in developing new methods to control the spread of tick-borne diseases.

**Here's a breakdown of the key findings from the "Results" section:**

**1. Increased Tick Burden on Infected Hosts:**

Field studies showed a significantly higher burden of *Haemaphysalis longicornis* ticks on *Apodemus agrarius* mice infected with SFTSV compared to uninfected mice.

This suggests that SFTSV infection in the mice may make them more attractive to ticks.

**2. Identification of Differential Volatile Metabolites (DVMs):**

Analysis of fecal and urine samples from infected mice revealed significant changes in the levels of various volatile metabolites.

Notably, 11 compounds were upregulated in fecal samples, including several indoles and phenols.

In urine samples, 29 compounds were downregulated, with eucalyptol and phenylalanine acid being the most prominent.

**3. Indole Compounds Attract Ticks:**

Y-tube Olfactometer: Ticks showed a significant preference for arms containing either SFTSV-infected mice or the specific indole compounds (indole and 3-methylindole) compared to control arms.

Electroantennography (EAD): Stimulation with both indole and 3-methylindole elicited significant electrophysiological responses in the tick's olfactory receptors.

**4. Identification and Characterization of Olfactory Proteins:**

Two olfactory-associated proteins were identified: NPC2 and OBPL.

NPC2: Predicted to have a β-barrel structure with a ligand-binding cavity.

OBPL: A classical OBP with a hydrophobic binding cavity.

Molecular docking analysis showed that both indole and 3-methylindole can bind to both NPC2 and OBPL.

Gene expression analysis revealed that OBPL gene expression increased in the tick's forelegs when exposed to indole and 3-methylindole, while NPC2 gene expression decreased.

**5. Functional Characterization of OBPL:**

Recombinant OBPL protein was successfully expressed and purified.

Binding assays demonstrated that OBPL has high affinity for both indole and 3-methylindole.

**Discussion Analysis:**

**Indoles and Host Health:** The authors highlight the complex role of indoles in host-microbe interactions. They emphasize that indoles can have both beneficial (e.g., reducing bacterial biofilm formation, modulating the immune system) and detrimental (e.g., potential mutagenicity) effects on the host. The upregulation of indoles in SFTSV-infected mice suggests a potential role in the host's defense response.

**Indoles as Olfactory Cues for Ticks:** The authors discuss previous research showing that indoles can act as attractants for various insects, including mosquitoes. This study extends this knowledge by demonstrating that indoles also attract ticks, specifically *Hae. longicornis*.

**Olfactory Mechanisms in Ticks:**  The authors acknowledge the lack of known olfactory receptors (ORs) in ticks, unlike in insects. They emphasize the importance of identifying and characterizing olfactory-associated proteins like NPC2 and OBPL in understanding tick olfaction. The study's findings suggest that OBPL plays a key role in the tick's perception of indole odors.

The biology of living organisms and the use of their behavior in control operations.

**Strengths:**

**Novelty**: The study provides new insights into the olfactory mechanisms underlying tick attraction to infected hosts. This is crucial for understanding tick-borne disease transmission.

**Rigorous Methodology**: The research employs a combination of field studies, laboratory experiments, and molecular analyses to support its conclusions.

**Clear Presentation:** The paper is generally well-written and easy to follow. The figures and tables are informative and effectively illustrate the key findings.

**Areas for Improvement:**

**Further Exploration of NPC2:** While the study focuses on OBPL, further investigation into the role of NPC2 in tick olfaction is warranted. Its downregulation in response to indole stimulation raises interesting questions about its potential function.

**Specificity of Indole Attraction:**  It would be valuable to explore whether the observed attraction to indoles is specific to SFTSV infection or if it extends to other pathogens that may also alter host odor profiles.

**Ecological Implications:** A more in-depth discussion of the ecological implications of these findings would be beneficial. How might this knowledge be used to develop novel tick control strategies?

**Overall:**

The research provides strong evidence that SFTSV infection in mice alters their odor profile, making them more attractive to *Haemaphysalis longicornis* ticks.

Indole and 3-methylindole appear to be key components of this "infection scent."

The OBPL protein plays a crucial role in the tick's perception of these indole odors.

**This finding suggests potential strategies for tick control by manipulating host odor or tick olfactory receptors.**

**This research sheds light on the complex interactions between ticks, hosts, and pathogens. Understanding these mechanisms could lead to novel methods for preventing tick-borne diseases like SFTSV.**

**References** Well Attributed, Latest Reference 2024, The number of references used in the research is 84. I think this is a large number. I suggest reducing the number of references, especially since there are a large number of them from previous, old years.

**In conclusion**, I recommend publishing this research in your important and discreet journal due to its scientific importance, after making the required modifications.

**Latakia, 12/01/2025**

**Reviewer**

**Prof. Dr. Nabil ABO KAF**
