# Supplementary material for: Smelly communication between haemaphysalis longicornis and infected hosts with indolic odorants: A case from severe fever with thrombocytopenia syndrome virus
Source: PLoS Negl Trop Dis. 2025 Jun 5;19(6):e0013139. doi: 10.1371/journal.pntd.0013139 (PMC12173412; doi:10.1371/journal.pntd.0013139)
Supplement: Attachment — Submitted filename: Response to Reviewers comments.docx [file pntd.0013139.s015.docx]

**Response to Reviewers’ comments**

1. **Responses to Editorial office**

**Dear editors:**

Thank you very much for the opportunity to improve our manuscript and your constructive suggestions that we had followed to update the present paper in a friendly manner.

Best wishes

Sincerely Yours

Yi Sun

**Q1**.	At this stage, the following Authors/Authors require contributions: Chunxiao Li.

**Response:** Done as your suggestions.

**Q2**.	Author Summary. This should appear in your manuscript between the Abstract (if applicable) and the Introduction, and should be 150-200 words long.

**Response:** Done as your suggestions.

Q3.	Copyright or trademark symbols - © on page: 29. - ® on page: 22.

**Response:** Done as your suggestions.

Q4.	Please upload all main figures as separate Figure files in .tif or .eps format.

**Response:** Done as your suggestions.

Q5.	Please add a full list of legends for your Supporting Information files after the references list.

**Response:** Done as your suggestions

Q6.	Data Availability Statement

**Response:** Done as your suggestions

Q7.	Please amend your detailed Financial Disclosure statement. "The funders had no role in study design, data collection and analysis, decision to publish, or preparation of the manuscript."

**Response:** Done as your suggestions

1) The file inventory includes files for Figures 1A,B, 4A,B, 5-1 to 5-4, 7A,B, 8A,C. We would recommend either combining these into a Figure 1,4,5,7,8 files with separate internal panels, or renumbering them as individual figures, as we are not able to publish multiple components of a single figure as separate files.
**Response:** Done as your suggestions

2) Thank you for stating" no competing interests" If you have no competing interests to declare, please state "The authors have declared that no competing interests exist". You may also provide an updated statement via email.

**Response:** Done as your suggestions

3) Please ensure that the Author List in your manuscript file matches the Author List in the online submission form exactly. Authors should be listed in the same order, and the order of individual first and last author names must be identical in both locations.

**Response:** Done as your suggestions

4) Thank you for stating "Ethical approval was granted by Beijing Institute of Microbiology & Epidemiology, AMMS Institutional Review Board, P. R. China. All experiments were performed in accordance with relevant guidelines and regulations.
Please include the full name of AMMS and also please include the approval number

**Response:** Done as your suggestions
5) We note that your Supplemental Figures are duplicated on your submission. Please remove any unnecessary or old files from your revision, and make sure that only those relevant to the current version of the manuscript are included.

**Response:** Done as your suggestions

6) Please confirm whether you drew the images / clip-art within the figure panels by hand.

**Response:** Yes. The image was designed and generated under the written permission from the copyright holder to publish the images or icons under CC BY 4.0 license.

2. **Response list**

Reviewer #2:

**Query 1**: Authors should include page numbers and use continuous line numbers to make it easier to locate the sentence.

**Response:** We indeed appreciated your suggestion and revised in the manuscript.

**Query 2** Page 2 Line 3: change “tick’s” to “ticks’”

**Response:** Done as your suggestion.

**Query 3** Page 2 Line 7: change “faces” to “fecal”

**Response:** Done as your suggestion.

**Query 4** Ensure consistency in abbreviations throughout the whole manuscript (e.g. Hae. longicorinis or H. longicorinis)

**Response:** Done as your suggestion.

**Query 5** Page 4 Line 12: make the sentence easier to understand (Thus,…)

**Response:** Thanks for your advices, we had rephrased the expression as “Consequently, the ticks of both strategies respond in a tactical and precise way to different olfactory cues from their respective hosts.”

**Query 6** Page 4 Line 15-17: is there any reference?

**Response:** We add a necessary reference (Sonenshine DE, Roe RM. Overview people, ticks, and animal. *In* Biology of Ticks, 2nd ed.; Sonenshine, D.E., Roe, R.M., Eds.; Oxford University Press: New York, NY, USA, 2014(1), pp. 3–16. Available at: https://digitalcommons.odu.edu/biology_books/1)

**Query 7** Page5 Line 16: “for the presence of” or “in the presence of”?

**Response:** Done as your suggestion.

**Query 8** At the end of the second part of the discussion, where does figure 9 come from? “…(Figure 9), we come to…”

**Response:** Sorry for my mistake, the figures had been renumbered and revised in the manuscript.

**Query** **9** In Supplemental Figure 2, is panel A totally same with panel B?

**Response:** Sorry for my fault, we misused the legends of supplemental figure 2 and now be updated in the revised manuscript.

**Query 10** Ensure consistency "Supplemental Figure SNo." or "Supplemental Figure No." throughout the whole manuscript

**Response:** We indeed appreciated your contributive advices and had revised our manuscript following your helps.

Reviewer #1:

**Query 1**. Abbreviations should be seriously checked throughout the paper. It is necessary to check whether *Hae. longicornis* is the correct abbreviation for *Haemaphysalis longicornis*. It is generally written as *H. longicornis* in many thesis, and two abbreviations are used interchangeably in this paper (*Hae. longicornis* & *H. longicornis*). Italics and abbreviations need to be unified, including the scientific names described in the data and data legend.

**Response:** Thanks for your information. We had revised the manuscript following your suggestions.

**Query 2.** There are various typos throughout the paper. Various typos were found, such as writing **feces as faces.** The overall content of the paper needs to be carefully checked and typos and grammatical errors need to be corrected.

**Response:** Done as your suggestions.

**Query 3**. The overall resolution of the data is poor. It would be desirable to revise this.

**Response:** Thanks for your information. We made a major revision to improve the resolution of our data.

**Query 4.** The Discussion section is somewhat long and the content is redundant. It would be better to concisely describe the key mechanisms and the possible contributions of the research results. In this regard, it would not be a bad idea to add visual materials that clearly summarize the experimental process and results.

**Response:** We indeed appreciate your suggestive advices. The part of discussion had been reorganized in the revised manuscript and a schematic graph (Figure 8) were also provided to summary our ideas.

Reviewer #:

**Query 1** The authors devoted significant space to discussing the relationship between microbiota and odor; however, this was not substantiated by the actual research findings. It is recommended to remove these redundant descriptions to enhance the conciseness of the article.

**Response:** Following your advices, we removed some redundant descriptions and rephrased our discussion to improve the conciseness of the revised manuscript (Page 19, line 550-578).

**Query 2** The authors did not adequately explain why indole or 3-methylindole was chosen as the focus of their research. After analyzing the data from the GC-MS, it appears that these two substances did not show the most significant increase. Why not choose other substances as subjects? Why were no other substances set as controls during the experiment? Did the author attempt to use the method described in the reference (López-López N et al., J Med Entomol. 2023;60(3):432-442. doi:10.1093/jme/tjad019) to evaluate synthetic compounds within alone, or in binary, tertiary, or quaternary mixtures? How did they determine whether indole or 3-methylindole has the best attraction effect? Merely stating that indoles are recognized as interspecies and inter-kingdom signal molecules is insufficient evidence.

**Response:** We did appreciate your advices and address our reasons to focus indole or 3-methylindole in the present research. Since indole or 3-methylindole, with its low molecular weight (< 300 Da) and high vapour pressure (0.01 kPa at 20°C), is known to be a quorum-sensing molecule signaling interspecies even interkingdoms, it met the criteria for a perceptible host VOC cue. Although these two substances did not show the most significant increase in our GC-MS analysis, their known signaling properties endow them high priority as potential semiochemicals. Therefore, we merely evaluated these compounds alone for their potential olfactometer responses to ticks as other contents are beyond our design. Thanks for your constructive advice, we had rephrased our expression in the revised manuscript (Page 15 line 427-431)

**Query 3** In the second part of the results, did the author test both indole and 3-methylindole to determine whether they have **synergistic, additive, or antagonistic** effects?

**Response:** No. The possible synergistic, additive, or antagonistic effects of indole and 3-methylindole would be beyond our design, and should be explored in future study.

**Query 4** In the fourth part of the results, it is insufficient to demonstrate the correlation between small molecule compounds and target genes based solely on transcriptional level validation. Additionally, from a data perspective, the changes observed are not very significant, ranging from 1.2 to 1.4-fold or as low as 0.6 to 0.8-fold. Is there any relevant literature that discusses the potential relationship between non-significant transcriptional expression changes in targets and compound interactions? If not, these findings could be included in supplementary materials or further explored through other approaches, such as examining translational levels or enzyme activity, etc.

**Response**: Thanks for your constructive advices. As the transcriptional expression changes of OBPL and NPC2 is merely a proxy of ligand–receptor interaction results, which did not comprehensively reflect the essential relationship between target compounds and potential protein receptors. It is reasonable to assume that OBPL was once an orphan receptor and that exogenous ligands were often associated with biological effects before the molecular identity of their target receptors was fully understood. In our study, differentially expressed genes of OBPL were identified with thresholds of |Log_2_FC|≥0.2, and P-value≤0.05 set significant differences. We achieved the significant changes of OBPL on transcriptional level, at least, exposed to high dose of exogenous ligands. To date, a mechanism known as deorphanization of receptors based on expression alteration of mRNA levels (DREAM) has been effectively employed in previous studies of the chemosensory-related proteins exposing to external odorant cues (He *et al.*, 2023, Pest Manag. Sci. 79 (4), 1410–1419. doi:10.1002/ps.7313; Anton and Rössler, 2021, Cell Tissue Res. 383 (1), 149–164. doi:10.1007/s00441-020-03329-z; Guerrieri et al., 2012, J. Exp. Biol. 215 (13), 2334–2341. doi:10.1242/jeb.060988). Therefore, we prefer to maintain the results and point out the limitation in our discussion.

**Query 5** On the other hand, if the conclusion in the fourth part of the results is valid, then NPC2 protein should be included as a control in the fifth part of the results. Have the authors attempted to validate the binding of the molecule and the protein using Microscale Thermophoresis (MST) experiments? These experiments provide a more accurate and credible assessment of protein-odor molecule interactions in an environment that is closer to the original conditions.

**Response**: Thanks for your constructive advices. We rebuilt our fluorescence competitive binding assays to validate the binding of the molecule and the protein with NPC2 protein as control and as expected, the NPC2 were merely demonstrated low affinity for indole (Ki 10.32μM) and 3-methylindole (Ki 15.36μM) respectively. Following your suggestion, we had updated our manuscript (Page 18 line529-545). Although Microscale thermophoresis (MST) is a reasonable biophysical assay used to quantify protein-protein and protein-drug interactions, the fluorescence competitive binding assays with F-380 fluorescence spectrophotometer would be sufficient to display the protein-odor molecule interactions with enough accurate and credible results. And we are willing to apply MST to the similar experiments in the coming future.

**Query 6** In the reference (Bunnell T, et al., J Chem Ecol. 2011;37(4):340-347. doi:10.1007/s10886-011-9936-1), an interesting phenomenon is reported. Specifically: 1) Sick hedgehogs tended to have elevated levels of indole in their feces. 2) Ticks were attracted to indole when given the option between indole and a solvent control. 3) Fecal matter from healthy hedgehogs, even with the addition of indole, did not attract ticks. However, the authors of this manuscript did not address the relationship between the aforementioned phenomenon and the experimental results presented in this study.

**Response:** Thanks for your information. We have noted the interesting reports by Bunnell and others in 2011, which also encourage us to explore the possible mechanism of the olfactory response of ticks. Following your advice, we welcome and address Bunnell's contributions, which are reported in the relevant part of the discussion in the revised manuscript. (Page 19 line 556-558，Page 21 line 594-602).
