# Supplementary material for: Smelly communication between haemaphysalis longicornis and infected hosts with indolic odorants: A case from severe fever with thrombocytopenia syndrome virus
Source: PLoS Negl Trop Dis. 2025 Jun 5;19(6):e0013139. doi: 10.1371/journal.pntd.0013139 (PMC12173412; doi:10.1371/journal.pntd.0013139)
Supplement: Attachment — Submitted filename: Response_to_reviewers_comments_auresp_2.docx [file pntd.0013139.s016.docx]

**Response to reviewer’s comments** **on** **PNTD-D-24-01605R1**

**Journal Requirements:**

Reviewer #2: [Figure 8]: Thank you for previously providing the copyright information for Figure 8. The GDP license is too restrictive compared to the PLOS CCBY 4.0 license, allowing for commercial reuse with attribution. Please either replace the images with CCBY 4.0 images or Public Domain or seek permission from the GDP team.

**Response:** We appreciate your advice and have decided to remove Figure 8 from our original manuscript for the sake of brevity.

**Conclusions**

Reviewer #2: The authors had a good discussion on the research results, but the focus of the discussion still needs to be carefully considered.

**Response:** Indeed, we appreciate your critical comments on the discussion of the manuscript. We have re-organized our discussion (see. Lines 555-590 on pages 19-20) to address the main points of the present manuscript.

**Editorial and Data Presentation Modifications?**

Reviewer #2:

1) line 92, 95: strategists or strategies?

**Response:** Done as your suggestions.

2) line 113: change to “sebaceous”

**Response:** Done as your suggestions.

3) line 139-140: First thing, this sentence did not elaborate on the relationship between VOCs and attracting vectors, plus, incorrect citation of literature. Is the content from Reference No. 25 actually? It seems that the citation order of most references is messy. Please ensure the correctness and citation order of the literature references.

**Response:** I apologise for my mistakes in the order of references, as we had followed your advice and checked the references carefully throughout the manuscript.

4) line 144: what dose “intense immature” mean?

**Response:** It should be “heavy immature….” and we had revised in the manuscript.

5) line 176: change "26 °C" to "26°C". Please ensure uniform format of measurement units throughout the whole manuscript. Pay attention spaces between numbers and units.

**Response:** Done as your suggestions.

6) line 187, 215, 216, 219…: same suggestion as above.

**Response:** Done as your suggestions.

7) line 273: what dose “EAG” mean?

**Response:** Done as your suggestions.

8) line 463: provide the full name: Microplusin-like (ML)

**Response:** Done as your suggestions.

9) line 466: change “Figure 4” to “Figure 3”

**Response:** Done as your suggestions.

10) line 524-525: under the stimulations of lower concentration

**Response:** Done as your suggestions.

11) line 934: Panel B, Panel C

**Response:** Done as your suggestions.
line 940-941: Italic “in vitro”

12) **Response:** Done as your suggestions.
13) line 947: The dissolution
    **Response:** Done as your suggestions.
14) line 974: change to “1-2”; “3:”
    **Response:** Done as your suggestions.
15) for Figure 4, authors should show more details in the pictures, i.e. key amino acid binding sites.

**Response:** Following your advices, we had updated the figure 4 with protein-ligand interaction prediction results

**Summary and General Comments**

Reviewer #2: Before acceptence, I still have identified a few minor issues with this paper.
1) This article focuses on the negative impact of tick-borne virus infection on tick feeding behavior. However, in the second paragraph of the discussion, this section is specifically devoted to explaining the benefits of indole metabolism on host health from an immune perspective, which confuses readers. Furthermore, in Figure 8, authors should emphasize current experimentally process and results.
**Response:** Indeed, we appreciate your critical comments on the discussion of the manuscript. We have re-organized our discussion (see. Lines 555-590 on pages 19-20) to address the main points of the present manuscript. We appreciate your advice and have decided to remove Figure 8 from our original manuscript for the sake of brevity.
2) Many typos, misuse of punctuation marks, incorrect citation order of references and inconsistent unit formats were still found. The whole manuscript including supplemental materials needs to be carefully checked.

**Response:** Thank you very much. We've gone through the whole manuscript carefully, and we've made changes in the revised version.
